# RNA Binding Protein Regulation and Cross-Talk in the Control of AU-rich mRNA Fate

**DOI:** 10.3389/fmolb.2017.00071

**Published:** 2017-10-23

**Authors:** Sofía M. García-Mauriño, Francisco Rivero-Rodríguez, Alejandro Velázquez-Cruz, Marian Hernández-Vellisca, Antonio Díaz-Quintana, Miguel A. De la Rosa, Irene Díaz-Moreno

**Affiliations:** Instituto de Investigaciones Químicas, Centro de Investigaciones Científicas Isla de la Cartuja, Universidad de Sevilla, Consejo Superior de Investigaciones Científicas, Seville, Spain

**Keywords:** mRNA fate, post-transcriptional regulation, RNA binding proteins, stability, translation

## Abstract

mRNA metabolism is tightly orchestrated by highly-regulated RNA Binding Proteins (RBPs) that determine mRNA fate, thereby influencing multiple cellular functions across biological contexts. Here, we review the interplay between six well-known RBPs (TTP, AUF-1, KSRP, HuR, TIA-1, and TIAR) that recognize AU-rich elements (AREs) at the 3′ untranslated regions of mRNAs, namely ARE-RBPs. Examples of the links between their cross-regulations and modulation of their targets are analyzed during mRNA processing, turnover, localization, and translational control. Furthermore, ARE recognition can be self-regulated by several factors that lead to the prevalence of one RBP over another. Consequently, we examine the factors that modulate the dynamics of those protein-RNA transient interactions to better understand the final consequences of the regulation mediated by ARE-RBPs. For instance, factors controlling the RBP isoforms, their conformational state or their post-translational modifications (PTMs) can strongly determine the fate of the protein-RNA complexes. Moreover, mRNA specific sequence and secondary structure or subtle environmental changes are also key determinants to take into account. To sum up, the whole understanding of such a fine tuned regulation is a challenge for future research and requires the integration of all the available structural and functional data by *in vivo, in vitro* and *in silico* approaches.

## Post-transcriptional regulation of gene expression by ARE-RBPs

**Figure 1:**
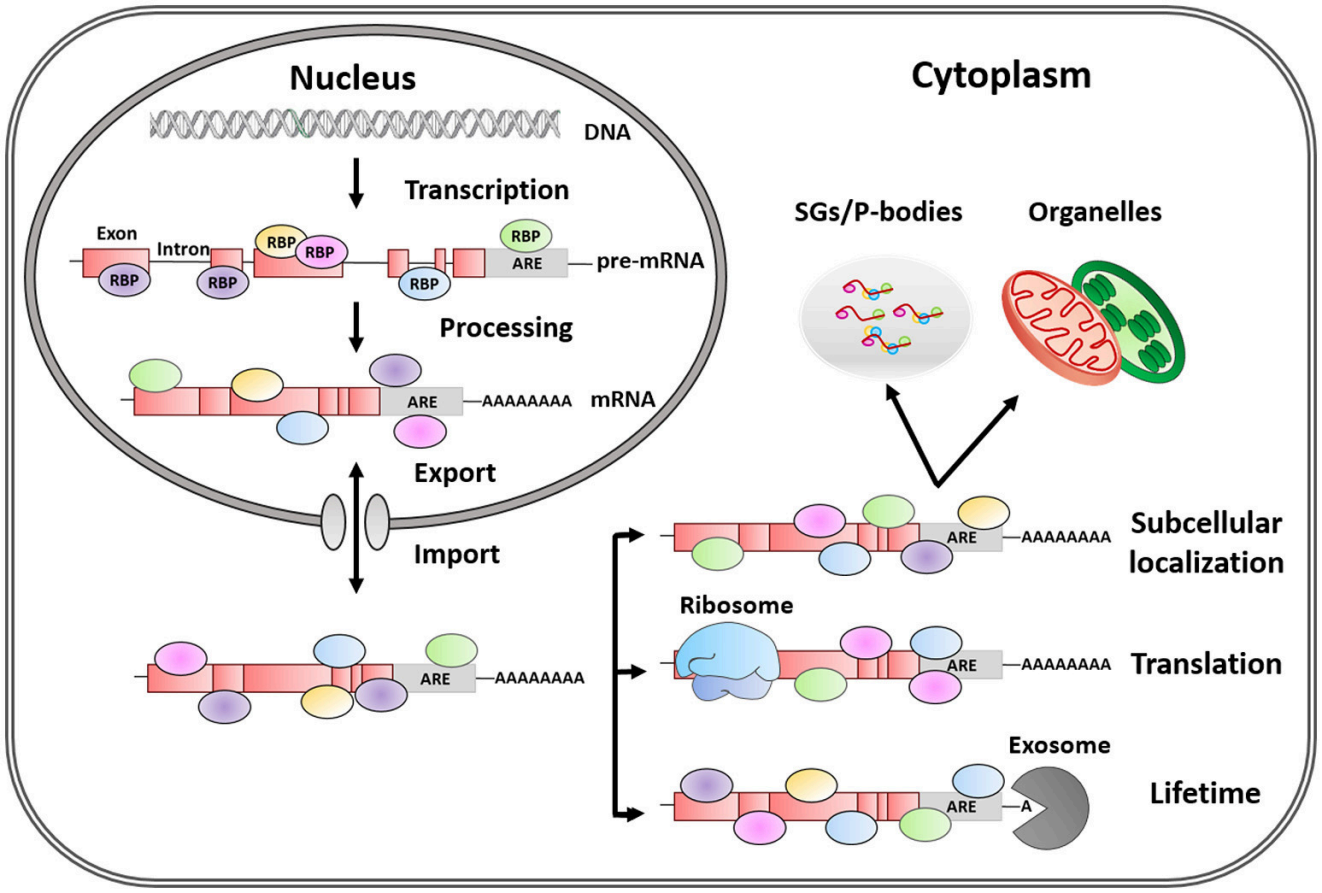
Post-transcriptional regulation of mRNA fate by RNA-binding proteins. RBPs are trans-acting elements, that shuttle between the nucleus and the cytoplasm, and influence mRNA fate by binding to regulatory sequences (cis-acting elements). AREs are the most common regulatory elements in 3′ UTR, and the binding of RBPs to these regions plays a key role in the life of mRNAs by regulating alternative mRNA splicing, maturation, transport, subcellular location, lifetime, and translation.

In eukaryotes, gene expression levels and protein abundance are often correlated but are subjected to a strict regulation. The control of mRNA metabolism allows cells to rapidly adapt to changing environmental conditions. Regulatory processes occurring after mRNA transcription—namely post-transcriptional control—strongly influence mRNA fate and, consequently, final protein levels (Vogel and Marcotte, 2012). Once mRNA transcription occurs in the nucleus, RNA Binding Proteins (RBPs) recognize the primary transcript or pre-mRNA to regulate its alternative splicing, polyadenylation, and capping (Figure 1). The generated mature mRNA is then transported to the cytoplasm by various other RBPs. Once in the cytoplasm, RBPs govern the stability, distribution to different cellular compartments and the translation of target mRNAs into their corresponding protein products (Matoulkova et al., 2012).

Within RBPs, ARE-RBPs function as trans-acting factors recognizing cis-acting elements in the 3′-Unstranslated Regions (UTR) of eukaryotic mRNA enriched in adenylate and uridylate (AU-rich elements or AREs). AREs are present in 5–8% of human genes with diverse functions such as cell growth and differentiation, signal transduction, apoptosis, nutrient transport, and metabolism. This list is dominated by genes involved in transient processes, which therefore require strict expression control (Barreau et al., 2005). For instance, the length and specific pattern of AREs may contribute to mRNA lifetime (Khabar, 2005). However, the final mRNA fate will be determined by the variable and dynamic ARE-RBPs/mRNA interactions or by RBP competition for the same transcript. Besides, ARE-RBPs bind to AREs via a variety of domains including the so-called RNA-Recognition Motif (RRM), the CCCH tandem zinc finger and the K-Homology domain (KH) (Stoecklin and Anderson, 2006; Clery et al., 2008; Valverde et al., 2008; Daubner et al., 2013). A single protein can contain several of these motifs leading to simultaneous interactions with either multiple targets or multiple sites within a particular target (Shen and Malter, 2015). Additionally, most ARE-RBPs shuttle between nucleus and cytoplasm; and their functions are linked to their specific subcellular distribution (Gama-Carvalho and Carmo-Fonseca, 2001).

In this mini-review we focus on the post-transcriptional regulation exerted by six of the best studied ARE-RBPs whose cross-talk has biological relevance and has been widely reported in the literature. Moreover, we examine the multiple intracellular signals and factors controlling the interactions between these proteins. AU-binding Factor 1 (AUF1), also known as Heterogeneous Nuclear RiboNucleo-Protein D (hnRNPD), is included for being the first identified ARE-RBP (Brewer, 1991). AUF1 is generally considered to promote the decay of target mRNAs, although the stabilization of some other transcripts has been also reported (Xu et al., 2001; Stoecklin and Anderson, 2006). Since AUF1 discovery, 20 additional ARE-RBPs have been identified. That list includes those that primarily promote mRNA degradation, such as Tristetraprolin (TTP) and KH domain-splicing regulatory protein (KSRP) (Gherzi et al., 2004; Sanduja et al., 2011); those stabilizing mRNA, such as Human antigen R (HuR) (Brennan and Steitz, 2001); and translational control proteins, such as T-cell intracellular antigen 1 (TIA-1) and TIA-1–related protein (TIAR) (Kawai et al., 2006; Mazan-Mamczarz et al., 2006).

## Interplay between ARE-RBPs in the post-transcriptional regulation of mRNAs

It is well-known that the substrates of post-transcriptional control are RNA ribonucleoprotein particles or RNPs containing mRNA molecules covered with RBPs, rather than naked mRNA (Szostak and Gebauer, 2013) (Figure 1). However, our understanding of how ARE-RBPs interact with each other at different regulatory levels is rather limited. Noticeably, some RBPs regulate the mRNA that encodes their own gene products, as well as those of other RBP counterparts, establishing self-regulatory loops controlling mRNA metabolism (Pullmann et al., 2007).

A good example of cross-talk between RBPs is the one involving HuR, KSRP and TTP proteins. These three proteins compete with each other for binding to common recognition sequences in the AREs that they regulate. Hence, TTP and KSRP negatively control the stability of several mRNAs—such as c-fos, TNFα and COX-2—whereas HuR generally acts in an opposite way, stabilizing them (Chen et al., 2001, 2002; Dean et al., 2001; Sawaoka et al., 2003; Katsanou et al., 2005; Winzen et al., 2007) with some exceptions (Katsanou et al., 2005; Kim et al., 2009) (Table 1). Moreover, TTP acts as a negative regulator of its own mRNA (Tchen et al., 2004; Lin et al., 2007) as well as HuR mRNA, its direct antagonist in mRNA regulation (Al-Ahmadi et al., 2009). On the other hand, HuR acts as a positive translational regulator of both KSRP and HuR mRNAs (Pullmann et al., 2007; Yi et al., 2010); while both proteins regulate the stability of their own mRNAs (Winzen et al., 2007; Al-Ahmadi et al., 2009) (Table 1, dashed square). Consequently, the redundant feedback involving KSRP, TTP, and HuR may provide a bi-stable signal transduction circuit in which either all or none of their target mRNAs are stabilized and/or translated. More intriguingly is the role of AUF1 in this regulatory loop as it presents four isoforms generated by alternative splicing of a single mRNA transcript (Wagner et al., 1998) with different RNA-binding affinities and specificities for its target mRNAs—such as c-fos, c-myc, TNFα, VEGF, and COX-2 (Brewer, 1991; Loflin et al., 1999; Lasa et al., 2000; Xu et al., 2001; Fellows et al., 2012).

Table 1Matrix representation of the interaction of selected RBPs (vertical axis) with the mRNA of those RBPs (Upper table) and several ARE-containing mRNA targets (Lower table, horizontal axis).

**Table:** 

	**mRNA**					
	**AUF1**	**TTP**	**KSRP**	**HuR**	**TIA-1**	**TIAR**
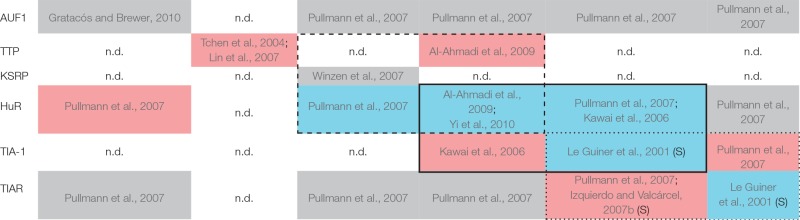

**Table:** 

	**ARE-containing mRNA**										
	**c-fos**		**c-myc**		**C*****c***	**TNFα**		**VEGF**		**COX-2**	
AUF1	Xu et al., 2001; Chen et al., 2002	Loflin et al., 1999	Xu et al., 2001; Liao et al., 2007	Brewer, 1991	n.d.	Xu et al., 2001		Fellows et al., 2012		Lasa et al., 2000^*^	
TTP	Chen et al., 2001		n.d.		n.d.	Lai et al., 1999; Chen et al., 2001; Lai and Blackshear, 2001		Lee et al., 2010		Sawaoka et al., 2003	
KSRP	Chen et al., 2001; Gherzi et al., 2004		Yamakoshi et al., 2010	Trabucchi et al., 2009	n.d.	Chen et al., 2001; Gherzi et al., 2004; Winzen et al., 2007		n.d.		Winzen et al., 2007	
HuR	Fan and Steitz, 1998; Peng et al., 1998; Chen et al., 2002		Kim et al., 2009		Kawai et al., 2006	Dean et al., 2001; Katsanou et al., 2005	Katsanou et al., 2005	Levy et al., 1998; Goldberg-Cohen et al., 2002		Sengupta et al., 2003; Katsanou et al., 2005	Katsanou et al., 2005
TIA-1	n.d.		Wang et al., 2010		Kawai et al., 2006	Piecyk et al., 2000		Hamdollah Zadeh et al., 2015	Hamdollah Zadeh et al., 2015	Dixon et al., 2003	
TIAR	n.d.		Liao et al., 2007		n.d.	Gueydan et al., 1999; Zhang et al., 2002^*^		Lu et al., 2009^*^		Cok et al., 2003	

Blue colors show positive regulation, whereas negative regulations are colored in red. Gray color indicates interactions that have been described but the effects were not examined. n.d., non-described; S, Splicing;

* *, Postulated regulations. The cross-talk between TTP, KSRP and HuR is highlighted by a dashed square; between HuR and TIA-1 by a black square; and between TIA-1 and TIAR by a dotted square*.

In addition to recognizing AU-rich sequences at the 3′ UTR of target mRNAs, some ARE-RBPs are able to activate splice 5′ sites followed by U-rich sequences. This is the case of TIA-1 and TIAR, that upregulate the translation of their own coding mRNAs (Le Guiner et al., 2001). Conversely, although consistent with their functional redundancy, their translation levels are negatively cross-regulated by each other (Le Guiner et al., 2001; Izquierdo and Valcárcel, 2007b; Pullmann et al., 2007) (Table 1, dotted square). Interestingly, TIA-1 and TIAR share common functions, acting as negative translational regulators of diverse mRNAs—such as c-myc, TNFα, VEGF, and COX-2—and are able to compensate for each other (Gueydan et al., 1999; Piecyk et al., 2000; Zhang et al., 2002; Cok et al., 2003; Dixon et al., 2003; Lu et al., 2009; Hamdollah Zadeh et al., 2015). In addition, it has been shown that HuR positively controls TIA-1 expression by enhancing its mRNA stability (Pullmann et al., 2007). By contrast, TIA-1 knockdown causes a marked increase in HuR levels, indicating that TIA-1 may contribute to lowering HuR levels in the cell (Kawai et al., 2006) (Table 1, black square). This is of great importance because both HuR and TIA-1 bind to cytochrome *c* (C*c*) mRNA, respectively promoting or inhibiting its translation without affecting its mRNA stability. The struggle between HuR (antiapoptotic factor) and TIA-1 (proapoptotic factor) for the control of C*c* mRNA translation underlies possible mechanisms to regulate both cellular respiration and programmed cell death. A direct binding between HuR and TIAR mRNA has also been reported (Pullmann et al., 2007) but, unexpectedly, TIAR does not seem to complex with C*c* mRNA, despite the extensively shared homology between TIAR and TIA-1 (Kawai et al., 2006).

## Factors that modulate ARE-RBP/mRNA interactions

Several examples of cross-talk between ARE-RBPs highlight that there must be an intricate network of regulatory events that lead to the prevalence of one RBP over the others when recognizing the same mRNA target. Thus, the regulatory activity of RBPs on gene expression is dynamic and adapts to cell conditions continuously. In this section, we briefly describe those factors for which there is evidence of their influence on the interaction between RBPs and their mRNA targets (Supplemental Figure 1).

### RBP isoforms

Alternative splicing is a highly regulated process that allows the synthesis of multiple different transcripts from the same gene, and is therefore an important source of protein diversity and complexity. The slight differences in amino acid sequence between isoforms can be determining for their function (Gallego-Páez et al., 2017). For example, TIA-1 and TIAR present two isoforms—*a* and *b*—in humans. Isoform *a* in TIA-1 and TIAR possesses 11 or 17 extra amino acids, respectively, that are critical for distinct functional properties. For instance, only TIAR isoform *a*—but not TIAR *b* and none of TIA-1 isoforms—has a translational silencing activity on the proteolytic enzyme Human Matrix Metalloproteinase-13 (HMMP13) in HEK293 cells. High levels of expression of HMMP13 have been documented in certain subset of cancers. Therefore, its downregulation by TIAR *a* may act as a tumor suppression mechanism (Yu et al., 2003).

As previously mentioned, AUF1 isoforms come from the alternative splicing of the same pre-mRNA. They differ as a function of the presence or absence of two independent domains encoded by exons 2 and 7. While p37^AUF1^ lacks both domains, p42^AUF1^ and p45^AUF1^ include a 49-amino acid domain encoded by exon 7 and p40^AUF1^ and p45^AUF1^ both contain a 19-amino acid domain encoded by exon 2. Inclusion of the exon 2-encoded sequence reduces the affinity of the first and second binding events of AUF1 dimers toward their mRNA substrates, but incorporation of the exon 7-encoded sequence increases the affinity of the second binding event. The isoform-specific differences provide unique biochemical characteristics that explain the diversity of AUF1 functions and complex regulation (Zucconi et al., 2010).

### RBP post-translational modifications

Post-Translational Modifications (PTMs), such as phosphorylation, isomerization, methylation, NEDDylation, acetylation, and ubiquitination of RBPs have a major influence on their function and/or their affinity toward their targets, with the consequent impact on mRNA stability, turnover and translation efficiency (Lee, 2012). For instance, the phosphorylation of p40^AUF1^ in residues Ser83 and Ser87 influences the sequential binding of dimers to TNFα mRNA (Wilson et al., 2003). Single phosphorylation of Ser83 inhibits by 40% the initial dimer binding to mRNA substrate, whereas Ser87-single phosphorylation induces a 2-fold increase in the affinity of the second binding event. In addition, when simultaneous phosphorylation of both residues occurs, the negative effect on the binding affinity of Ser83 prevails over the positive effect of Ser87 (Wilson et al., 2003). Several phosphorylation sites have also been identified in TTP (Cao et al., 2006, 2014). Phosphorylated TTP binds with a lower affinity than the dephosphorylated TTP to target AREs (Carballo et al., 2001; Hitti et al., 2006). Phosphorylation of RBPs can also modify their activity without altering the affinity for mRNA targets. Such is the case of TIA-1 and TIAR, whose splicing control over the *Fas* gene sequence determines the expression of the pro-apoptotic membrane-bound form in detriment of the anti-apoptotic soluble one (Izquierdo and Valcárcel, 2007a). Moreover, HuR methylation has been proposed to increase the nuclear export of HuR, which could be important for mRNA localization (Li et al., 2002). NEDDylation of HuR increases its stability and lifetime, which, in turn, can affect the total levels of HuR target mRNAs due to its main stabilizing action (Embade et al., 2012; Fernández-Ramos and Martínez-Chantar, 2015).

### RBP conformational changes

ARE-RBPs can undergo conformational changes upon binding to their targets (Ellis and Jones, 2008). These variations can be detected in the contact surface with mRNAs as well as in distant areas, meaning that ARE-RBPs can adapt both the local and global structure. An example of conformational changes that influence ARE recognition has been reported for KSRP. An inter-domain re-arrangement, that orients the two central KH domains and their RNA-binding surfaces creating a two-domain unit, is crucial for its role in ARE-mediated mRNA decay (Supplemental Figure 2) (Díaz-Moreno et al., 2010). Additionally, some of the PTMs mentioned above can also influence the conformation of RBPs. Hence, the phosphorylation of Ser193 within the N-terminal KH motif (KH1) of KSRP leads to the unfolding of this structurally atypical and unstable domain, creating a binding site for 14-3-3ζ, driving the nuclear localization of KSRP and controlling its mRNA-degradation activity (Díaz-Moreno et al., 2009).

Another important regulation factor is the RBP oligomerization state upon mRNA recognition. HuR RRM1 domain and RRM1-2 di-domain (the main platform of cytoplasmic mRNA binding in HuR) form homodimers in solution (Benoit et al., 2010). This phenomenon is dependent on Cys13, which is able to form disulfide bonds. Such homodimerization may modulate HuR function upon oxidative stress. Moreover, the HuR RRM3 domain has been found to be involved in protein oligomerization and RNA recognition, both functions regulated by the same RRM but using different surfaces at opposite sides of the domain. The conserved Trp261 residue is key for dimerization, as the substitution by glutamic acid alters its dimerization dynamics and stabilizes the monomeric state (Scheiba et al., 2014; Díaz-Quintana et al., 2015).

### Cellular conditions and stress response

Eukaryotic cells have evolved sophisticated strategies to overcome stress. One of them is the assembly of Stress Granules (SGs), which allows mRNA translation silencing and protection from degradation. Among RBPs with critical roles in neurodegenerative diseases, TIA-1 proteins are essential in SG formation (Mazan-Mamczarz et al., 2006; Vanderweyde et al., 2012). Hence, under hypoxic conditions, TIA-1 and TIAR block the expression of hypoxia-inducible factor (HIF)-1α through binding to its ARE-containing mRNA (Gottschald et al., 2010). Inhibition of this transcription factor is enhanced when both RBPs are organized into SGs. In addition, HuR also aggregates into SGs to halt the translation of specific housekeeping mRNAs under stress conditions (Bergalet et al., 2011). The deregulation of SGs results in cytoplasmic accumulation and subsequent pathologies such as Parkinson and Alzheimer (Vanderweyde et al., 2012).

Variations in pH values can also modulate the binding of TIA-1 to nucleic acids, acting as a pH-dependent molecular switch. The p*K*_a_ values of the histidine imidazole groups of TIA-1 RRM2 and RRM3 are substantially higher in complexes with short RNA and DNA oligonucleotides than in the isolated domains. Interestingly, those p*K*_a_ values are also controlled by slight environmental pH changes (Cruz-Gallardo et al., 2013, 2015). This fact provides valuable information to understand the pH effect on ARE-RBPs when shuttling among cellular compartments with different pHs (nucleus, cytoplasm, SGs, etc.).

During oxidative stress, AUF1 binding to mRNAs containing 8-oxo-7,8-dihydro-guanine could play a role in the selective elimination of oxidized mRNA by presumably driving their degradation (Ishii et al., 2015). Finally, HuR localization can also be altered upon different stress signals such us UV, actinomycin D or hydrogen peroxide, leading to the cytoplasmic accumulation of the protein. However, after a heat shock treatment, the decrease in HuR protein levels enhances cell survival. This phenomenon is linked to the ubiquitination of Lys182, promoting protein degradation, which finally interferes with the binding of HuR to its target mRNAs (Abdelmohsen et al., 2009).

### mRNA specific sequence and conformation

RBPs do not interact with the same affinity with every ARE-containing mRNA; instead, preferences exist for certain sequences. For instance, TIA-1 RRM domains display different binding constants during nucleic acid recognition. Indeed, the central domains (RRM2 and RRM3) constitute the mRNA binding platform of the protein. RRM2 drives the interaction with RNA, and shows the highest affinities for pyrimidine rich sequences. In turn, RRM3 enhances the overall TIA-1 binding affinity for RNA, preferentially interacting with C-rich motifs (Cruz-Gallardo et al., 2014; Wang et al., 2014; Waris et al., 2017). Moreover, HuR and TIAR interact with U- and AU-rich mRNAs *in vitro*, with greater affinity (≈10-fold) for the former ones. This higher affinity for U-rich mRNAs results from a higher association rate constant, mainly derived from the presence of a greater number of effective binding positions (Kim et al., 2011). However, *in vivo* analysis showed that HuR stabilized AU-rich mRNAs to a greater extent than U-rich mRNAs (Brennan and Steitz, 2001). Additionally, the KH domains of KSRP behave as independent binding modules with different affinities for AU-rich mRNAs, explaining the broad range of targets recognized by the protein. While the fourth KH domain (KH4) is primarily responsible for mRNA binding and decay through an essential structural element in its β_4_, KH3 is also necessary to drive the recognition of AU- and G-rich sequences. On the other hand, all KH domains show a clear negative selection for C-rich sequences (García-Mayoral et al., 2007, 2008). Interestingly, many RNA targets of HuR, which acts antagonistically to KSRP, often contain isolated Gs but very rarely Cs (López De Silanes et al., 2004).

Conformational changes in the ARE-mRNA structure have also the potential to regulate the binding affinity of RBPs. These changes may precede the binding of RBPs, as occurs with TNFα mRNA as a consequence of the stabilization of its folding mediated by divalent cations such as Mg^2+^ (Wilson et al., 2001a,b). In addition, the AU-rich motif of TNFα mRNA can also adopt a hairpin-like structure that inhibits specifically p37^AUF1^ binding, but hardly affects its interaction with HuR (Fialcowitz et al., 2005). On the other hand, the association of RBPs can cause local changes in the structure of their cognate mRNAs, which may affect the recruitment of new *trans*-acting factors or establish preferences for one RBP over another. Consequently, these changes would directly impact on the turnover rates of such ARE-containing mRNAs (Wilson et al., 2001b; Zucconi et al., 2010).

### DNA recognition and role of RBPs in DNA damage response

Some ARE-RBPs also have the ability to bind to DNA. Importantly, in the case of TIA-1 and TIAR, it occurs with a markedly higher affinity than both RBPs show for their mRNA targets (Suswam et al., 2005; Waris et al., 2017). In fact, it has been hypothesized that the formation of the RBP-mRNA complexes would require the direct displacement of the RBP from its DNA-binding site by the polymerase. This dual binding capacity of TIA-1 and TIAR could be potentially providing a link between transcription and splicing (Suswam et al., 2005; Mcalinden et al., 2007; Waris et al., 2017).

Interestingly, several RBPs are involved in DNA Damage Response (DDR), being recruited to DNA breaks in a Poly (ADP-Ribose) (PAR)-dependent manner and/or forming liquid-like compartments by phase separation (Kai, 2016). The formation of these phases requires the presence of an unstructured Prion-Related Domain (PRD) like the one that is present in TIA-1 and TIAR proteins (Gilks et al., 2004). Importantly, abnormal phase separation by mutated PRD-containing proteins leads to pathological protein aggregation and is associated with neurodegenerative and aging-associated diseases (Kai, 2016).

## Conclusions and future perspectives

Before being translated into proteins, mRNAs are subjected to a sequential and strict control by RBPs exerted by the recognition of AREs in their 3′-UTRs. Regulation of mRNA homeostasis through ARE-RBPs allows the fine tuning of responses by controlling mRNA translation, degradation, or storage in diverse eukaryotic cell compartments (Glisovic et al., 2008; Ganguly et al., 2016). As reviewed above, many examples of ARE-RBP interactions have been reported in the literature, but it is still not well-understood how RBP domains collaborate or compete with each other for the modulation of its targets. The proper inspection of such a convoluted interplay between RBPs requires the combination of different methods in order to compensate the specific strengths and weaknesses of each technique. On the other hand, it becomes more and more evident the need of a transition from a static to a dynamic point of view to take into account the biological environment during RNA binding. Consequently, the integration of the information obtained by *in vivo* approaches with the structural data would be of great interest. Moreover, the understanding of the ARE-mRNAs processing in highly dynamic and often transient macromolecular complexes also remains challenging (Rissland, 2017). Finally, the key role of intrinsically disordered connecting linkers between RNA binding domains has acquired significant relevance in the latest reports (Basu and Bahadur, 2016). Altogether, the examples of mRNA-protein interactions by ARE-RBPs herein reviewed highlight the need for integrative studies to fully understand such a fine tuned regulation.

## Author contributions

All authors listed have made a substantial, direct, and intellectual contribution to the work, and approved it for publication.

### Conflict of interest statement

The authors declare that the research was conducted in the absence of any commercial or financial relationships that could be construed as a potential conflict of interest. The reviewer SM-L and handling Editor declared their shared affiliation.

## Abbreviations

AREs: AU-Rich Elements
ARE-RBPs: RNA Binding Proteins that recognize AU-Rich Elements
AUF-1: AU-binding Factor 1
c-fos: Finkel-Biskis-Jinkins murine osteosarcoma viral oncogene homolog
c-myc: Avian myelocytomatosis virus oncogene cellular homolog
COX-2: Cyclooxygenase-2
DDR: DNA Damage Response
hnRNPD: Heterogeneous Nuclear RiboNucleo- Protein D
HuR: Human antigen R
KH: hnRNP K-Homology
KSRP: KH type Splicing Regulatory Protein
PAR: Poly (ADP-Ribose)
P-bodies: Processing bodies
PTMs: Post-Translational Modifications
PRD: Prion-Related Domain
RBPs: RNA Binding Proteins
RNPs: Ribonucleoprotein Particles
RRM: RNA Recognition Motif
SGs: Stress Granules
TIA-1: T-cell Intracellular Antigen 1
TIAR: TIA-1 Related protein
TNFα: Tumor Necrosis Factor α
TTP: Tristetraprolin
UTR: Untranslated Region
VEGF: Vascular Endothelial Growth Factor.
