# Supplementary material for: RNA Binding Protein Regulation and Cross-Talk in the Control of AU-rich mRNA Fate
Source: Front Mol Biosci. 2017 Oct 23;4:71. doi: 10.3389/fmolb.2017.00071 (PMC5660096; doi:10.3389/fmolb.2017.00071)
Supplement: Supplementary file 1 [file DataSheet1.doc]

**Supplemental Information**

**RNA Binding Protein regulation and cross-talk in the control of AU-rich mRNA fate**

Sofía M. García-Mauriño, Francisco Rivero-Rodríguez, Alejandro Velázquez-Cruz, Marian Hernández-Vellisca, Antonio Díaz-Quintana, Miguel A. De la Rosa and Irene Díaz-Moreno


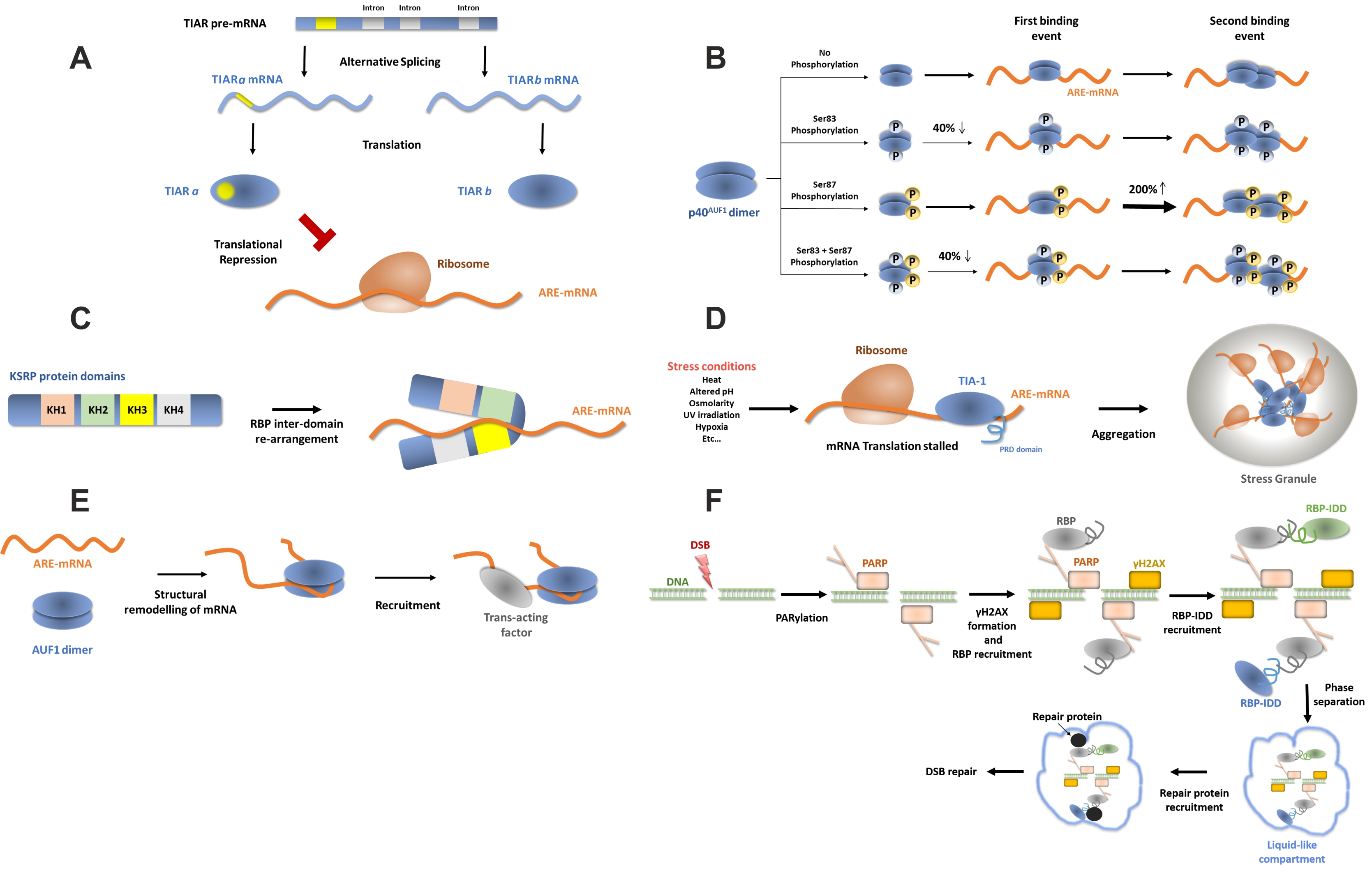


**Supplemental Figure 1. Factors that modulate ARE-RBP/mRNA interactions. A.** **RBP isoforms**. TIAR *a* and TIAR *b* isoforms come from the alternative splicing of the same pre-mRNA. The extra 11 amino acids in isoform *a* (highlighted in yellow) allows TIAR *a* to exert a translational repression effect on HMMP13 mRNA (Yu et al., 2003). **B. RBP post-translational modifications.** Phosphorylation of p40^AUF1^ influences the sequential binding of dimers to TNFα mRNA. Single phosphorylation at Ser83 inhibits by 40% the initial binding of dimers whereas Ser87-single phosphorylation induces a 2-fold increase in the affinity of the second binding of dimers. The effect of Ser83 prevails to that of Ser87 when both residues become phosphorylated (Wilson et al., 2003). **C. RBP conformational changes.** Upon ARE recognition, KSRP experiences an inter-domain re-arrangement that orients the two central KH domains (KH2 and KH3) and their RNA-binding surfaces creating a two-domain unit that its crucial for its role in mRNA decay (Díaz-Moreno et al., 2010). **D. Cellular conditions and stress response.** Under stress conditions, TIA-1 blocks the translation of ARE containing mRNAs leading to protein aggregation and stress granules formation through its PRD domains. This mechanism protects target mRNAs from degradation and is critical in neurodegenerative disorders (Vanderweyde et al., 2012). **E. mRNA conformation.** Association of AUF1 to its cognate RNA substrates may be responsible for its ability to remodel local RNA structures and subsequent recruitment of additional trans-acting factors (Wilson et al., 2001). **F**. **Role of RBPs in DNA damage response.** Upon DNA double-strand break (DSB), RBPs are recruited to DNA damage sites in a PARP-dependent manner. This step allows γH2AX formation. The association of intrinsically disordered domains (IDD) from RBPs forms liquid-like compartments or liquid-demixing. Finally, this phase separation allows the recruitment and action of DSB repair proteins (Kai et al., 2016).


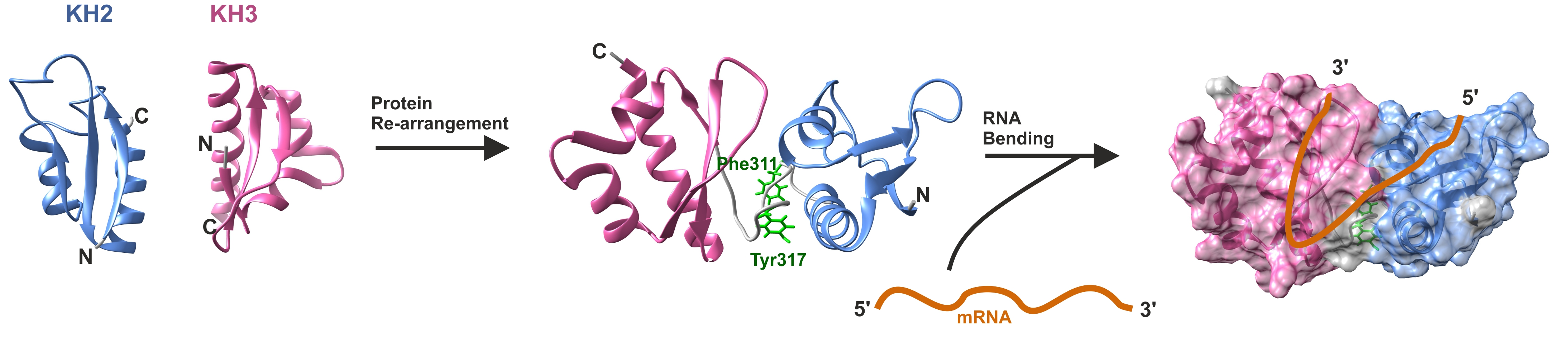


**Supplemental Figure 2. KH2 and KH3 inter-domain arrangement in KSRP is important for the interaction with its mRNA targets.** Ribbon representation of the KH2 and KH3 single domains (*left*) or the KH23 di-domain (*center*). Surface representation of the KH23 structure of KSRP (*right*). KH2 is represented in blue, KH3 in magenta and the linker between the domains in grey. The side-chains of Phe311 and Tyr317 (key residues in the domain–domain contacts) are shown in green. The di-domain re-arrangement is crucial for the specific interaction of the protein with its AU-containing mRNA targets (depicted in orange), which must undergo a bend to properly bind to KSRP (Díaz-Moreno et al., 2010).

**References**

Díaz-Moreno, I., Hollingworth, D., Kelly, G., Martin, S., García-Mayoral, M., Briata, P., Gherzi, R., and Ramos, A. (2010). Orientation of the central domains of KSRP and its implications for the interaction with the RNA targets. *Nucleic Acids Res.* 38**,** 5193-5205. doi: 10.1093/nar/gkq216

Kai, M. (2016). Roles of RNA-Binding Proteins in DNA damage response. *Int. J. Mol. Sci.* 17**,** 310. doi: 10.3390/ijms17030310.

Vanderweyde, T., Yu, H., Varnum, M., Liu-Yesucevitz, L., Citro, A., Ikezu, T., Duff, K., and Wolozin, B. (2012). Contrasting pathology of the stress granule proteins TIA-1 and G3BP in tauopathies. *J. Neurosci.* 32**,** 8270-8283. doi: 10.1523/jneurosci.1592-12.2012

Wilson, G.M., Lu, J., Sutphen, K., Suarez, Y., Sinha, S., Brewer, B., Villanueva-Feliciano, E.C., Ysla, R.M., Charles, S., and Brewer, G. (2003). Phosphorylation of p40AUF1 regulates binding to A + U-rich mRNA-destabilizing elements and protein-induced changes in ribonucleoprotein structure. *J. Biol. Chem.* 278**,** 33039-33048. doi: 10.1074/jbc.M305775200

Wilson, G.M., Sutphen, K., Moutafis, M., Sinha, S., and Brewer, G. (2001). Structural remodeling of an A + U-rich RNA element by cation or AUF1 binding. *J. Biol. Chem.* 276**,** 38400-38409. doi: 10.1074/jbc.M106509200

Yu, Q., Cok, S.J., Zeng, C., and Morrison, A.R. (2003). Translational repression of human matrix metalloproteinases-13 by an alternatively spliced form of T-cell-restricted intracellular antigen-related protein (TIAR). *J. Biol. Chem.* 278**,** 1579-1584. doi: 10.1074/jbc.M203526200
